# Celiac in the twenty-first century—the change in BMI of children at diagnosis over the last two decades

**DOI:** 10.1007/s00431-024-05835-6

**Published:** 2024-12-26

**Authors:** Rim Kasem Ali Sliman, Nili Stein, Yigal Elenberg Alter

**Affiliations:** 1 Technion - Israel Institute of Technology, Haifa, Israel; 2 Department of Pediatrics, Clalit Health Care Organization, Carmel Medical Center, Haifa, Israel; 3 Pediatric Gastroenterology Unit, Carmel Medical Center, Haifa, Israel; 4 Data Research and Statistician Center, Clalit Health Care Organization, Carmel Medical Center, Haifa, Israel

**Keywords:** Celiac, BMI, Pediatrics, Obesity, Overweight, Underweight

## Abstract

This study examined the prevalence of different BMI categories among newly diagnosed pediatric celiac disease (CD) patients in Israel from 2002 to 2018. A retrospective cross-sectional study using the database of Clalit Health Services in Israel included 5520 newly diagnosed CD children aged 2–18 between 2002 and 2018. Data on BMI, gender, ethnicity, and socioeconomic status (SES) were collected and analyzed Of the 5520 CD patients, 57.5% were female, 39.7% had low SES, and 77.1% were Jewish. At diagnosis, 13.1% were underweight, 73% had normal BMI, 9.1% overweight, and 4.8% were obese. Underweight and obese patients tended to be older at diagnosis (9 years) compared with normal and overweight patients (8 years) (*P* < 0.001). A higher proportion of Arab patients were underweight, while more Jewish patients were obese. Lower SES was significantly associated with increased underweight risk (*P* < 0.001). Over time, diagnosed patients had improved SES and were less underweight (*P* < 0.001). Male gender increased obesity risk (OR 1.36 [95% CI 1.06–1.74], *P* = 0.017), while Arab ethnicity was protective for obesity (OR 0.67 [95% CI, (0.45–0.99)], *P* = 0.047)

*Conclusion*: Underweight prevalence significantly decreased in the second decade, but no significant change in overweight and obesity was noted. Underweight was associated with older age at diagnosis, poverty, and Arab ethnicity. Obesity was associated with older age and was more frequent in Jewish and male patients. Physicians should have a low threshold for CD screening regardless of BMI status to enable timely diagnosis and treatment to prevent long-term health consequences.

**Table:** 

**What Is Known:** *• Celiac disease is traditionally associated with underweight due to malabsorption, but recent reports suggest an increasing prevalence of overweight and obesity in pediatric patients at diagnosis* **What Is New:** *• This study found that underweight prevalence decreased significantly over time, while overweight and obesity prevalence remained unchanged. Underweight was associated with older age, poverty, and Arab ethnicity, while obesity was more common in Jewish and male patients*

**Supplementary Information:**

The online version contains supplementary material available at 10.1007/s00431-024-05835-6.

## Introduction

Celiac disease (CD) is a lifelong condition affecting the small intestine in genetically predisposed individuals [1]. It is one of the most prevalent chronic childhood diseases, affecting approximately 0.5–3% of the Western population [2].

It is characterized by an autoimmune response triggered by gluten ingestion, damaging the small intestinal lining [2, 3]. It is marked by the presence of antibodies such as serum anti-tissue transglutaminase (tTGAb) and anti-endomysium autoantibodies (EMAs) [4]. Additionally, characteristic alterations are observed in small bowel biopsies, including lymphocyte infiltration, crypt hyperplasia, and villous atrophy, as per the Marsh-Oberhuber classification. Currently, the sole viable and efficacious treatment option is a lifelong gluten-free diet (GFD) [4].

The disease can emerge at any point in life and displays heterogeneous clinical expressions. While the classic presentation entails symptoms like diarrhea, abdominal distention, malnutrition, and failure to thrive, it seems to have changed in recent decades. The proportions of patients with classical gastrointestinal symptoms, such as weight loss, diarrhea, and malabsorption, are decreasing in adults and children. In contrast, atypical manifestations have become more prevalent [1–3, 5].

Children with CD may present with diverse symptoms. Notably, a remarkable proportion of patients, including children, may exhibit very mild symptoms or be entirely asymptomatic. In such cases, the disease can only be detected through active screening of high-risk groups [1, 6]. Very young ones frequently display “classic” signs like diarrhea, abdominal distension, and growth retardation, while abdominal pain, vomiting, and constipation are atypical and more common in older children and teenagers [1]. Additionally, CD diagnosis in children can be carried out if extra-intestinal manifestations such as arthritis, neurological disorders, anemia, recurrent aphthous stomatitis, osteopenia, delayed puberty, and amenorrhea, are present, and when screening a child or adolescent with celiac disease-associated conditions [1, 3, 5, 7].

Traditionally, CD is expected to cause nutrient malabsorption and lead to poor weight gain, weight loss, and undernutrition due to its impact on the small intestinal mucosa. However, recent reports have highlighted an increasing prevalence of overweight or obesity in adults, adolescents, and children at the time of initial CD diagnosis. This trend mirrors the rising rates of overweight and obesity in the general population of many developed countries [5, 8]. Notably, obesity is the predominant nutritional concern among children and adolescents in the USA and Europe [4]. A recent Israeli study found a 10.8% overweight prevalence and 15% obesity prevalence among children “https://www.health.gov.il/UnitsOffice/ICDC/HealthAndLifestyle/Overweight_Obesity/Pages/Obesity_motoring.aspx.”.

The prevalence of overweight/obese pediatric patients at the time of CD diagnosis is unknown. The existing literature mentions that CD coexists with overweight/obesity in some reports [4]. Our objective was to examine the prevalence of different BMI categories among a substantial group of newly diagnosed pediatric CD patients from 2002 to 2018 and analyze changes over time.

## Methods

This study is based on data from electronic medical files of new CD patients diagnosed between 2 and 18 years old, which were recorded on the computerized database of Clalit Health Services in Israel. We retrieved all CD patients diagnosed from 01.01.2002 to 31.12.2018 with a BMI measured ± 3 months from diagnosis.

Additional data collected were: sex, age at the diagnosis, and socioeconomic status (SES).

SES (low, middle, high) were based on the SES scores of the clinic neighborhood as defined by the Israeli Central Bureau of Statistics.

BMI is calculated as weight (kg) divided by height (m) squared. Underweight (< 5%), normal (5–85%), overweight (85–95%), and obesity (> 95%) classifications by the World Health Organization (WHO) definitions according to the percentile of BMI for age and sex “https://www.who.int/news-room/fact-sheets/detail/obesity-and-overweight”.

We excluded other diagnoses that could impact BMI, such as type 1 diabetes, hypothyroidism, Cushing’s disease, Crohn’s disease, ulcerative colitis, SLE, and rheumatoid arthritis.

Our study is a retrospective cross-sectional study, authorized by the Institutional Review Board (Helsinki committee), approval number 0108–19-CMC.

### Statistical methods

Continuous variables were summarized with mean ± standard deviation, median, and IQR. Categorical variables were presented as numbers and proportions.

Differences in demographical characteristics among the four groups of BMIs were analyzed using the chi-square test for the categorical variables and one-way ANOVA or Kruskal–Wallis, as appropriate, followed by an independent *t*-test or Mann–Whitney for pairwise comparisons with Bonferroni correction.

Changes in demographical characteristics between the two time periods were analyzed using the chi-square test for the categorical variables and the independent *t*-test or Mann–Whitney for the continuous variables. Logistic regression was used to analyze the association between BMI at the time of diagnosis and the two time periods adjusted for age, gender, SES, and ethnicity in three different models, underweight, overweight, and obese, with normal BMI as the reference group. OR with a 95% confidence interval is shown.

All statistical analyses were performed using IBM SPSS Statistics 28.0 (IBM, New York, NY). For all analyses, *P* < 0.05 for the 2-tailed tests was considered to be statistically significant.

## Results

A total of 10,564 CD pediatric patients aged 2 to 18 years old were diagnosed between 2002 and 2018; for 5753 of them, BMI 3 months within diagnosis was documented. After exclusion, a total of 5520 patients were included in our study (Supplementary Material 1), with 1471 cases from 2002 to 2010 and 4049 cases from 2011 to 2018. At diagnosis, 4029 (73%) had normal BMI, 722 (13.1%) were underweight, 503 (9.1%) were overweight, and 266 (4.8%) were obese, as shown in Table 1.

**Table 1:** Demographic data divided into BMI categories

		**BMI**				**Total (*N* = 5520)**
		**Underweight** **(*N* = 722)**	**Normal (*N* = 4029)**	**Overweight (*N* = 503)**	**Obese (*N* = 266)**	
**Age at diagnosis**		9.08 ± 4.1	7.66 ± 4.2	8.0 ± 4.5	9.0 ± 4.6	7.94 ± 4.2
**Gender**	**Males**	343 (47.5%)	1679 (41.7%)	194 (38.6%)	129 (48.5%)	2345 (42.5%)
	**Females**	379 (52.5%)	2350 (58.3%)	309 (61.4%)	137 (51.5%)	3175 (57.5%%)
**SES**	**Low**	369 (51.1%)	1558 (38.7%)	172 (34.2%)	92 (34.6%)	2191 (39.7%)
	**Medium**	216 (29.9%)	1376 (34.2%)	196 (39%)	100 (37.6%)	1888 (34.2%)
	**High**	132 (18.3%)	1080 (26.8%)	134 (26.6%)	73 (27.4%)	1419 (25.7%)
	**Missing**	5 (0.07%)	15 (0.04%)	1 (0.2%)	1 (0.4%)	22 (0.4%)
**Ethnicity**	**Jews**	492 (68.1%)	3119 (77.4%)	422 (83.9%)	221 (83.1%)	4254 (77.1%)
	**Arabs**	230 (31.9%)	910 (22.6%)	81 (16.1%)	45 (16.9%)	1266 (22.9%)

*BMI* body mass index, *SES* socioeconomic status

The mean age at diagnosis for all patients was 7.94 ± 4.2 years. Patients with normal and overweight BMI were diagnosed at 7.66 ± 4.2 and 8 ± 4.5 years, respectively, while underweight and obese patients were diagnosed around a year later at 9.08 ± 4.2 and 9 ± 4.6 years (*P* < 0.001). Regarding gender, 42.5% of patients were males, and most patients of both genders had a normal BMI, 71.6% of the males and 74% of the females (Table 1).

The analysis revealed a statistically significant association between SES and BMI category (*P*-value < 0.001). It was observed that patients with lower SES had the lowest BMI, comprising 51% of all underweight CD patients. This finding suggests that having a lower SES increases the prevalence of being an underweight CD patient at diagnosis (Table 1).

The majority of the patients were Jews (77.1%), which is similar to the population distribution in Israel (74% Jews and 21% Arab). However, there was a notable difference in the proportion of Arabs across the BMI categories. The underweight BMI group had the highest percentage of Arab patients at 31%, compared with only 16.9% in the obese group (*P*-value < 0.001) (Table 1).

A comparison between the 2002–2010 and 2011–2018 time periods revealed several significant differences, as shown in Table 2. The mean age at diagnosis decreased from 8.3 ± 4.5 years in the earlier period to 7.8 ± 4.1 years in the later period (*P* < 0.001). Additionally, there was an improvement in SES over the years, with the percentage of patients with low SES decreasing from 47.5% in 2002–2010 to 37.0% in 2011–2018 (*P* < 0.001). The proportion of Jewish patients diagnosed increased from 69.2 to 79.9% over the decades (*P* < 0.001), aligning more closely with the ethnic distribution in Israel (74% Jewish, 21% Arab). Furthermore, the distribution of BMI categories showed a statistically significant difference (*P* < 0.001) between the two time periods, with significantly fewer underweight cases diagnosed in the later period after Bonferroni correction compared with normal (Table 2).

**Table 2:** Demographic data by years

		_**Year of diagnosis**_		_**Total**_ **(*N* = 5520)**	_***P*-value**_
		_**2002–2010**_	_**2011–2018**_		
_**Age at diagnosis (years)**_		_8.3±4.5_	_7.82±4.1_	_7.94±4.2_	_<0.001_
_**Gender**_	_**Males**_	_43.1%(634)_	_42.3%(1711)_	_42.5% (2345)_	_0.575_
	_**Females**_	_56.9% (837)_	_57.7% (2338)_	_57.5% (3175)_	
_**SES**_	_**Low**_	_698 (47.5%)_	_1493 (37%)_	_2191 (39.9%)_	_<0.001_
	_**Medium**_	_463 (31.5%)_	_1425 (35.4%)_	_1888 (34.3%)_	
	_**High**_	_307 (20.9%)_	_1112 (27.6%)_	_1419 (25.8%)_	
_**Ethnicity**_	_**Jews**_	_1018 (69.2%)_	_3236 (79.9%)_	_4254 (77.1%)_	_<0.001_
	_**Arabs**_	_453 (30.8%)_	_813 (20.1%)_	_1266 (22.9%)_	
_**BMI**_	_**Underweight**_	_241 (16.4%)_	_481 (11.9%)_	_722 (13.1%)_	_<0.001_
	_**Normal**_	_1051 (71.4%)_	_2978 (73.5%)_	_4029 (73%)_	
	_**Overweight**_	_113 (7.7%)_	_390 (9.6%)_	_503 (9.1%)_	
	_**Obese**_	_66 (4.5%)_	_200 (4.9%)_	_266 (4.8%)_	

*BMI* body mass index, *SES* socioeconomic status

Three analyses were conducted to compare different BMI categories in CD patients upon diagnosis. First, when comparing underweight to normal BMI (excluding overweight and obese), increasing age increased the chance of being diagnosed as an underweight CD patient (OR 1.08 [95% CI 1.06–1.10], *P* < 0.001). Boys had a higher risk of being diagnosed as underweight CD patients compared with girls (OR 1.27 [95% CI 1.08–1.49], *P* = 0.003). Additionally, low SES was a risk factor for being an underweight CD patient at diagnosis compared with middle and high SES (*P* < 0.006, *P* < 0.001 accordingly). Over time, the chance of being diagnosed as an underweight CD patient decreased, with patients in the second period being less underweight compared with the first period (OR 0.77 [95% CI 0.65–0.92], *P* = 0.001). However, ethnicity did not show any statistical significance in relation to underweight status at diagnosis (Table 3).

**Table 3:** The relationship between the year of diagnosis and the different BMI categories of celiac disease patients at diagnosis, adjusted for demographic factors

		**Underweight compared with normal (*N* = 4751)**		**Overweight compared with normal (*N* = 4798)**		**Obese compared with normal** **(*N* = 4295)**	
		**OR (95% CI)**	***P*-value**	**OR (95% CI)**	***P*-value**	**OR (95% CI)**	***P*-value**
**Age**		1.08 (1.06–1.10)	< 0.001	1.02 (0.99–1.04)	0.060	1.08 (1.05–1.11)	< 0.001
**Boys vs. girls**		1.27 (1.08–1.49)	0.003	0.89 (0.73–1.07)	0.216	1.36 (1.06–1.74)	0.017
**Arabs vs. Jews**		1.14 (0.91–1.41)	0.257	0.66 (0.49 –0.89)	0.006	0.67 (0.45–0.99)	0.047
**SES**	**Low**	**Ref**	**Ref**	**Ref**	**Ref**	**Ref**	**Ref**
	**Middle**	0.74 (0.59–0.92)	0.006	1.07 (0.83–1.37)	0.597	1.04 (0.74–1.46)	0.825
	**High**	0.61 (0.47–0.78)	< 0.001	0.91 (0.69–1.20)	0.502	0.99 (0.69–1.43)	0.958
**Celiac year diagnosis**	**2002–2010**	**Ref**	**Ref**	**Ref**	**Ref**	**Ref**	**Ref**
	**2011–2018**	0.77 (0.65–0.92)	0.001	1.18 (0.95–1.48)	0.143	1.06 (0.79–1.41)	0.712

*BMI* body mass index, *SES* socioeconomic status, *OR* odds ratio, *CI* confidence interval, *Ref* reference

The second analysis compared overweight to normal BMI CD patients at diagnosis (excluding underweight and obese). Ethnicity was the only factor that showed a statistically significant association. When controlling for other factors, being an Arab was associated with a lower likelihood of being overweight at diagnosis (OR 0.66 [95% CI 0.49–0.89], *P* = 0.006) compared with normal BMI (Table 3).

The third analysis compared obese to normal BMI CD patients (excluding underweight and overweight). Increasing age was significantly associated with a higher likelihood of obesity at diagnosis (OR 1.08 [95% CI 1.05–1.11], *P* < 0.001). Furthermore, being male emerged as a risk factor for being diagnosed as an obese CD patient (OR 1.36 [95% CI 1.06–1.74], *P* = 0.017). In addition, Arab ethnicity decreased the chance of being diagnosed as an obese CD patient (OR 0.67 [95% CI (0.45–0.99)], *P* = 0.047) (Table 3).

## Discussion

Classically, CD presents with gastrointestinal symptoms like diarrhea, abdominal pain, cramping or bloating, failure to thrive, weight loss, irritability, and weakness; an increasing number of children exhibit delayed onset, atypical manifestations, or even asymptomatic. These can include minimal intestinal complaints like nausea, vomiting, or even constipation, along with extra-intestinal manifestations such as short stature, delayed puberty, iron deficiency anemia, and other nutritional deficiencies [3, 5, 9].

In recent decades, CD prevalence has increased in children, challenging traditional views of the condition. Notably, there’s a rising proportion of overweight and obese patients at diagnosis, contrasting with the historical presentation of malabsorption and growth failure [10–12]. This shift may be attributable to an improved understanding of disease genetics and immune features, better recognition of atypical presentations, expanded serological testing and screening of at-risk asymptomatic groups, with an actual rise in prevalence [10, 12]. A Finnish study reported that CD prevalence doubled across sexes and age groups during the period examined, aligning with observations from large screening studies in the USA and Europe. This trend occurs in the context of increasing rates of other autoimmune and allergic diseases in developed countries, including type 1 diabetes, multiple sclerosis, and Crohn’s disease [7, 13]. The “hygiene hypothesis” offers a thought-provoking explanation for the rise in autoimmune diseases like celiac. It suggests that the main factor underlying the increased prevalence of autoimmune diseases is the reduction in the incidence of infectious diseases. An early childhood infection or normal establishment of Indigenous intestinal microbiota could downregulate immunity and suppress different autoimmune disorders [7, 14–16]. Other theories include the growing number and compliance of pediatric vaccines given during early childhood., recurrent early-life antibiotic use, and consumption of highly processed Western foods [17–22].

The 2012 ESPGHAN guidelines for CD diagnosis shifted towards serological testing and away from endoscopic diagnosis [6, 23]. In Israel, as elsewhere, this change led to an increase in CD diagnosis rates, with nearly three times more patients identified in the second period of our study. This surge can be attributed to both a potential rise in CD prevalence and increased diagnostic rates due to simplified testing procedures. The new approach allows healthcare providers to order serological tests even for patients with low clinical suspicion, potentially capturing cases that might have been missed under previous protocols. Ultimately, this shift has fundamentally altered the landscape of CD diagnosis from a specialized procedure to a more routine and accessible part of healthcare.

Our study aligns with previous findings, revealing that 73% of pediatric CD patients had a normal BMI, 13.1% were underweight, 9.1% were overweight, and 4.8% were obese at diagnosis. These results further reinforce the notion that CD occurs across different weight categories, including overweight and obesity, in the pediatric population.

Furthermore, our study revealed that obese and underweight CD patients were generally older at diagnosis (mean age 9 years) compared with overweight (mean age 8 years) and normal BMI patients (mean age 7.6 years). This contrasts with another study suggesting overweight/obese children were slightly younger at diagnosis, though the difference was insignificant [8]. Our findings may indicate a potential diagnostic delay for obese CD patients, as excess weight gain and obesity are unexpected and atypical presentations associated with the intestinal mucosal damage caused by CD.

Additionally, most CD patients were females (57.5%), consistently across all BMI categories. However, males had a higher risk of being underweight or obese at CD diagnosis compared with females, while other studies reported conflicting evidence [8, 24].

Regarding SES, most patients had lower SES, and those with lower SES had the lowest BMI. This finding suggests that having a lower SES increases the prevalence of being an underweight CD patient at diagnosis. Additionally, the proportion of Jews and Arabs diagnosed with CD was in accordance with the general population distribution in Israel. The higher underweight prevalence among Arab CD patients might indicate delayed diagnosis until severe manifestations like significant weight loss become apparent. In contrast, the higher overweight/obesity among Jewish patients could stem from socioeconomic factors and diagnostic bias, wherein clinicians may overlook CD in overweight or obese individuals. This can be explained by the fact that lower SES can increase the prevalence of being underweight due to limited resources and dietary options. On the other hand, lower SES populations often experience delayed diagnosis and other conditions due to poverty-related barriers to accessing healthcare services [22, 25, 26]. These findings warrant further research to explore the underlying factors contributing to ethnic differences in disease presentation, diagnosis, and referral patterns, such as healthcare access barriers or cultural influences impacting timely referrals of Arab patients. Addressing these potential disparities is crucial for improving early CD identification and management across diverse populations.

Several theories may explain the increasing prevalence of overweight/obesity in pediatric celiac disease patients. The “compensation theory” suggests that unabsorbed nutrients from proximal intestinal villous atrophy get compensatory absorbed distally, preventing weight loss, and patients may also become overweight/obese, like anybody else [4, 8, 27]. Another theory is the emerging evidence of the roles of epigenetics, gut microbiota, and specific disease pathways contributing to this phenomenon. Additionally, the rising overweight and obesity trends in the general pediatric population and early diagnosis before malnutrition occurs may also be reflected in celiac patients [12, 28].

While obesity is a known presentation in adults with CD, affecting 0.45 to 39% at diagnosis [4, 8], it is a rare and atypical presentation in children [29]. Recent studies indicate that over 50% of newly diagnosed pediatric CD patients have a normal BMI, with prevalence rates of obesity ranging from 5 to 21% [2, 8, 30]. Multiple studies across different countries like the USA, Sweden, and England corroborate these findings, with normal BMI rates in CD children ranging from 66 to 74.5% [2], “https://www.who.int/news-room/fact-sheets/detail/obesity-and-overweight”. Interestingly, CD prevalence among overweight/obese children varied, ranging from 3.4 to 5%, with one study reporting 4% prevalence in Italian children referred to an auxological clinic, while an English study found 28% overweight and 5% obese among CD patients, and in an Iranian study, it was 3.4% [4, 8], “https://www.who.int/news-room/fact-sheets/detail/obesity-and-overweight”, [12, 21]. These findings consistently demonstrate that normal BMI is common in pediatric CD, emphasizing that BMI is not a reliable predictor of CD in children and underscoring the need for other diagnostic criteria [2, 8, 31].

In summary, the year of celiac disease diagnosis did not show a statistically significant association with being overweight or obese, while there is a significant relationship with decreasing the chances of being an underweight CD patient at diagnosis. However, other demographic factors such as age, gender, ethnicity, and SES were considered, and their associations with overweight were evaluated. Arabs and female gender had significantly lower odds of being obese compared with Jews.

Further in-depth research is needed to explore potential contributing factors influencing the association between ethnicity and overweight/obesity status in CD patients. Understanding sociocultural, economic, and healthcare access influences is crucial to address these disparities and improve diagnostic practices and management strategies across different populations, ultimately leading to better outcomes for all CD patients.

## Conclusion

In conclusion, CD prevalence has grown over the years. In obese and overweight children with non-specific gastrointestinal complaints, in the appropriate clinical setting, physicians should consider CD as one of the differential diagnosis since it can be easily missed. Failure to do so may lead to unnecessary diagnostic delay and place the children at risk for long-term adverse health consequences.

Furthermore, our findings suggest that older age and male gender increased the risk of obesity at CD diagnosis. At the same time, Arab ethnicity was associated with a lower risk of obesity compared with normal BMI. Additionally, patients with lower SES were more likely to be underweight CD at diagnosis.

## Supplementary Information

Below is the link to the electronic supplementary material.

Supplementary file1 (JPG 61 KB)

Supplementary file2 (PPTX 39 KB)

## Data Availability

No datasets were generated or analysed during the current study.

## Abbreviations

BMI: Body mass index
CD: Celiac disease
EMA: Anti-endomysium autoantibodies
GFD: Gluten-free diet
GGT: Gamma-glutamyl transferase
GOT: Glutamate oxaloacetate transaminase
GPT: Glutamate pyruvate transaminase
HDL: High-density lipoprotein
LDL: Low-density lipoprotein
SES: Socioeconomic status
SLE: Systemic lupus erythematosus
T4: Thyroxine
tTGAb: Anti-tissue transglutaminase antibodies
TSH: Thyroid-stimulating hormone
WHO: World Health Organization
