# Supplementary figures and images for: Celiac in the twenty-first century—the change in BMI of children at diagnosis over the last two decades
Source: Eur J Pediatr. 2024 Dec 26;184(1):105. doi: 10.1007/s00431-024-05835-6 (PMC11669609; doi:10.1007/s00431-024-05835-6)

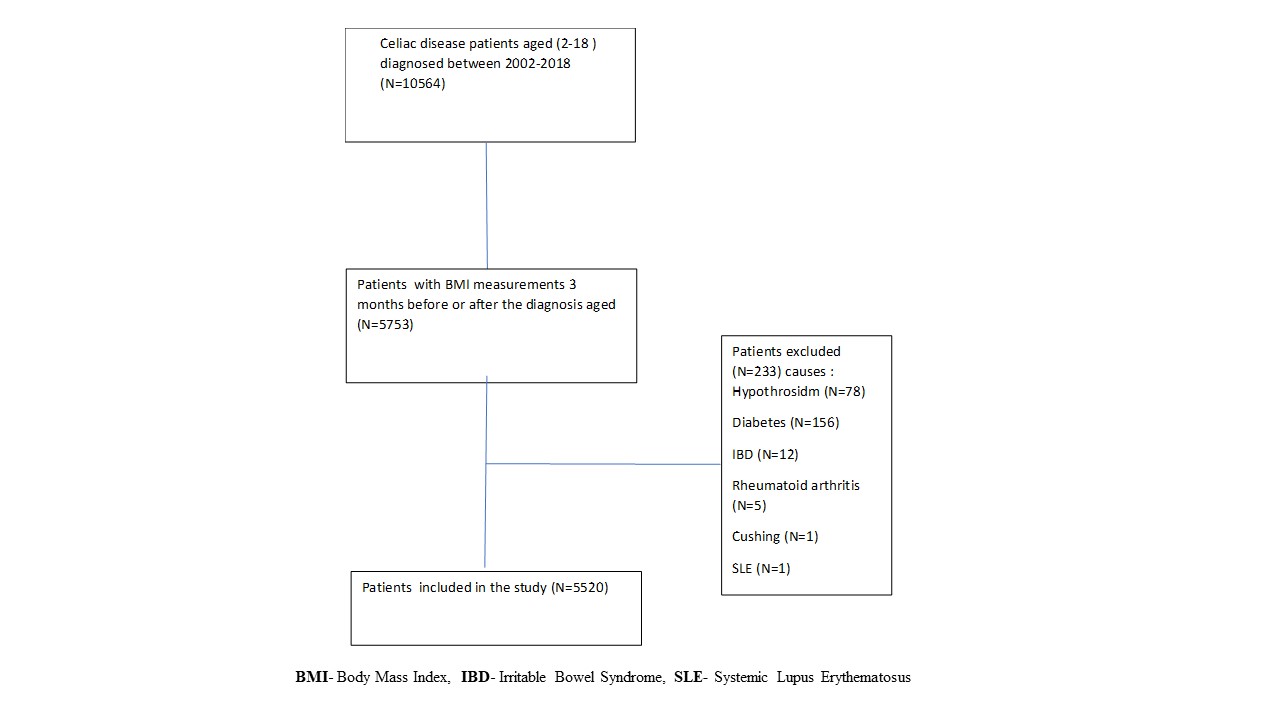

Supplement: Supplementary file 1 — Supplementary file1 (JPG 61 KB) [file 431_2024_5835_MOESM1_ESM.jpg]

## Slide 1
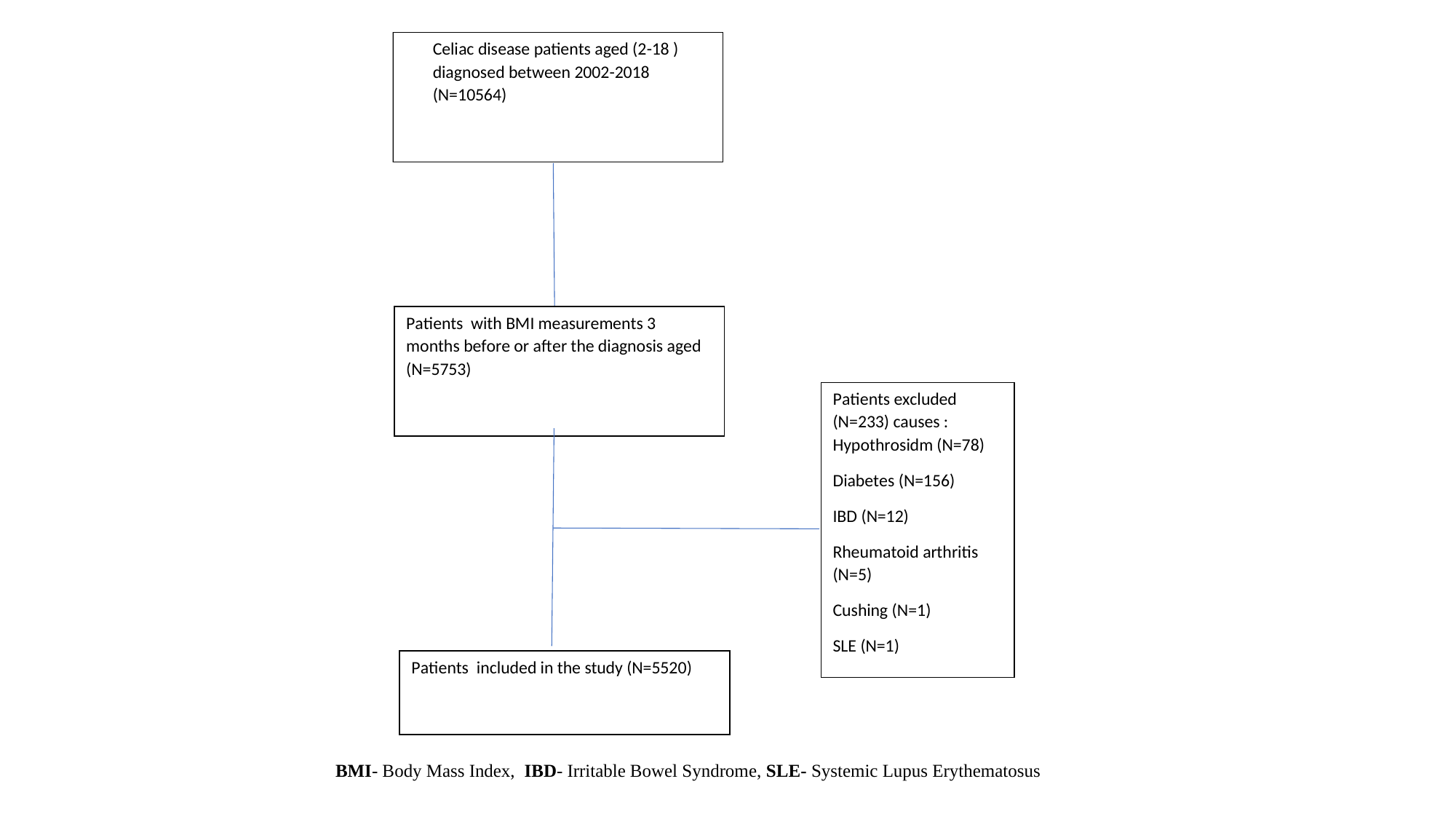

BMI- Body Mass Index, IBD- Irritable Bowel Syndrome, SLE- Systemic Lupus Erythematosus

Supplement: Supplementary file 2 — Supplementary file2 (PPTX 39 KB) [file 431_2024_5835_MOESM2_ESM.pptx]
